# Breast imaging findings in haematological malignancies

**DOI:** 10.1007/s13244-014-0344-2

**Published:** 2014-08-07

**Authors:** K. N. Glazebrook, S. Zingula, K. N. Jones, R. T. Fazzio

**Affiliations:** Department of Radiology, Mayo Clinic, 200 First St SW, Rochester, MN 55905 USA

**Keywords:** Mammography, Ultrasound, PET, CT, Haematological malignancy

## Abstract

**Objectives:**

The objectives of this article are to review and illustrate the imaging appearances of haematological malignancies in the breast.

**Methods:**

With Institutional Review Board approval, a search of the surgical pathology records from 1st January 2000 to 1st July 2012 was performed for haematological malignancies.

**Results:**

Forty-eight cases of haematological malignancies (42 women and 6 men) were identified with imaging available for review: 39 cases of breast lymphoma, 6 cases of chronic lymphocytic leukaemia, 2 cases of acute leukaemia and 1 case of known multiple myeloma.

**Conclusions:**

Breast manifestations of haematological malignancies are rare. They can have a variable appearance at imaging and can mimic primary breast carcinoma. In the setting of suspicious breast imaging findings, pathological diagnosis of haematological malignancy is concordant. Correlation with a clinical history of prior haematological malignancy can be helpful in suggesting the diagnosis and help prevent unnecessary surgical treatment.

***Teaching Points*:**

• *Breast haematological malignancies are rare but the imaging appearances can mimic breast carcinoma*.

• *Breast lymphoma, most often B*-*cell non*-*Hodgkin lymphoma, may be primary or due to secondary disease*.

• *At ultrasound, haematological malignancies may present as a heterogeneous or predominantly echogenic mass*.

• *Haematological malignancies show intense activity on PET*/*CT except myeloma which has low FDG uptake*.

## Introduction

Haematological malignancies are common, accounting for approximately 9 % of new cancer diagnoses in the United States in 2013 [1]. However, manifestations of these malignancies in the breast are rare [2]. A prior history of haematological malignancy may be helpful in diagnosing secondary breast lesions; however, the breast presentation may be the initial diagnosis of a systemic process [3]. The purpose of this article is to review the imaging findings of haematological malignancies in the breast which can mimic primary breast cancer.

With Institutional Review Board approval, a search of surgical pathology records of approximately 12,000 breast biopsies performed at our institution from 1st January 2000 to 1st July 2012 yielded 48 cases of haematological malignancies; 39 cases of breast lymphoma (19 representing primary lymphoma and 20 with known secondary lymphoma, median age 66 years, range 30–89 years), 6 cases of chronic lymphocytic leukaemia (4 representing a new diagnosis and 2 with known chronic lymphocytic leukaemia [CLL], median age 68 years, range 62–76 years), 2 cases of acute leukaemia (1 representing a new diagnosis and 1 with known leukaemia, ages 31 and 83 years) and 1 case of known multiple myeloma (age 57 years). The majority of patients presented with palpable findings on self or clinical breast examination (*n* = 40); the remaining patients having a lesion identified at screening mammography or incidentally found on computed tomography (CT). The majority of cases were female (42 women and 6 men). Mammograms were available in 45 patients and breast ultrasound in 46 patients. Positron emission tomography/computed tomography (PET/CT) images were also available for six patients. None of the patients received a dedicated breast magnetic resonance imaging (MRI) examination, presumably because diagnosis of haematological malignancy (via image-guided percutaneous biopsy) negated the need for this form of advanced imaging. All imaging studies were reviewed by fellowship-trained radiologists specialising in breast imaging and intervention (K.G., R.F., S.Z., K.J.). Here we review and illustrate the salient imaging features of haematological malignancies in the breast.

## Breast lymphoma

Breast lymphoma (BL) may occur as either a primary breast tumour or as an extranodal manifestation of secondary disease. By definition, primary breast lymphoma first manifests in the breast without evidence of lymphoma elsewhere, except ipsilateral axillary and supraclavicular lymph nodes [2, 4]. Additionally, there should be no prior history of lymphoma. Extranodal disease refers to lymphomatous involvement of tissues other than lymph nodes. Almost any organ can be affected; however, common extranodal sites include the stomach, Waldeyer ring, central nervous system, lung, bone and skin [5].

Primary breast lymphoma accounts for 0.85–2.2 % of all extranodal lymphomas and 0.1–0.5 % of breast neoplasms [6, 7]. Secondary lymphoma is slightly more common than primary lymphoma. The World Health Organisation classification for breast tumours subdivides lymphomas of the breast into diffuse large cell lymphoma, Burkitt lymphoma, extranodal marginal-zone B-cell lymphoma of MALT type and follicular lymphoma. Primary breast lymphomas are usually non-Hodgkin type with B-cell lineage [8]. Some forms of T-cell lymphoma are more common in Asia than in Western countries [2].

Clinically, BL most commonly presents as enlarging, painless breast mass(es), although pain can be present in up to 25 % of cases. Less common presentations include nipple retraction or discharge and diffuse skin thickening and oedema, mimicking inflammatory breast cancer [8]. Burkitt lymphoma is more common in black women and may present as massive, bilateral breast enlargement [8]. B type symptoms of sweating, weight loss and fever are rare in primary BL but more commonly noted in patients with secondary BL. Age range can be wide but the median age is 55–65 years [8, 9]. In our series there was a wide age range (30–89 years) with a median age of 66 years.

### Mammography

**Fig. 1:**
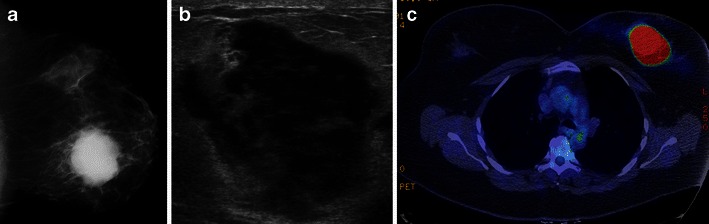
A 45-year-old woman with primary B-cell lymphoma presents with a palpable mass in the left breast. **a** Left cranio-caudal mammogram demonstrates a high-density irregular mass with indistinct margins in the medial left breast. **b** Ultrasound of the palpable mass demonstrates an irregular hypoechoic mass with thin echogenic rim. **c** Axial PET/CT shows homogeneous intense hypermetabolic activity within the mass. No uptake is seen within normal appearing nodes in the left axilla (*not shown*)

**Fig. 2:**
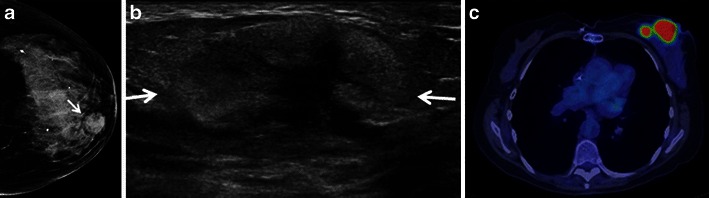
A 74-year-old woman with primary large B cell breast lymphoma presenting with a palpable mass in the left breast. **a** Cranio-caudal left breast mammogram shows an isodense circumscribed mass (*arrow*) in the medial left breast. **b** Ultrasound of the palpable mass demonstrates an oval, circumscribed principally echogenic mass with an irregular shaped central area of hypoechogenicity (*arrows*). **c** PET/CT shows two areas of homogeneous hypermetabolic uptake in the medial left breast. No axillary lymph node uptake was seen (*not shown*)

**Fig. 3:**
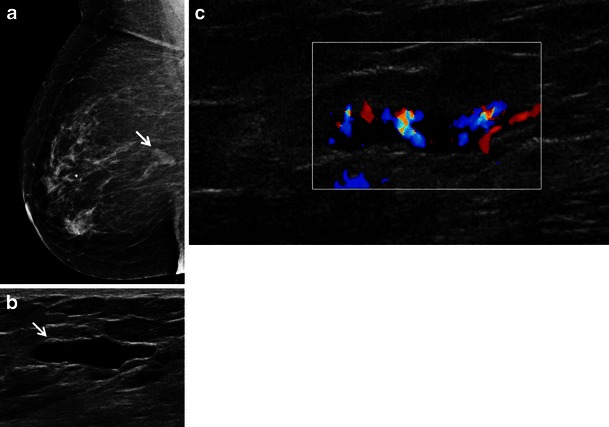
A 67-year-old woman with a history of marginal zone B-cell lymphoma (mucosa associated lymphoid tissue [MALT] lymphoma) presents with screening mammogram detected new mass in the right breast. **a** Right medio-lateral oblique mammogram demonstrates a lobulated circumscribed isodense mass in the upper right breast posterior depth (*arrow*). **b**, **c** Ultrasound shows an anechoic pseudocystic mass without posterior enhancement (*arrow*) which shows marked increased vascularity on colour Doppler evaluation indicating this is a solid mass

**Fig. 4:**
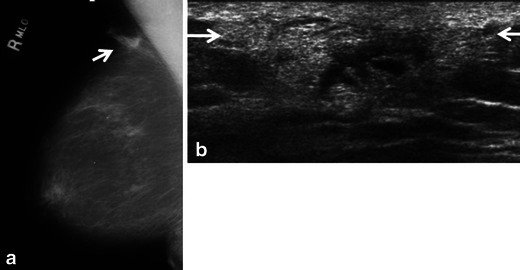
A 72-year-old woman with marginal zone B-cell lymphoma with follicular colonisation presents with developing focal asymmetry within the right upper breast noted on screening mammogram. **a**. Ill-defined focal asymmetry noted on the right medio-lateral oblique screening mammogram (*arrow*). **b** Ultrasound shows an irregular principally hyperechoic mass (*arrows*) extending to involve the skin with central areas of hypoechogenicity

BL may be identified incidentally on screening mammograms [7, 8]. Solitary non-calcified masses represent the most common reported mammographic finding in BL [6, 7]. Lesions may be bilateral in up to 28 % [6]. Margin analysis is less consistent. In a contemporary series reported by Surov et al. [6], margins were circumscribed in 66 % of cases, lobulated in 30 % and indistinct in 3 %. However, indistinct margins were noted in 72 % of cases in a series reported by Yang et al. [8]. Spiculated margins are rarely demonstrated. BL presenting as architectural distortion is also rare. In our series, approximately half of all BL cases represented irregular masses with indistinct margins (Fig. 1), and one-third demonstrated circumscribed margins (Figs. 2 and 3). Four cases presented as a developing asymmetry (Fig. 4), and one case as architectural distortion. Diffuse infiltration by lymphomatous deposits may also occur but may be obscured at mammography in patients with dense breast tissue. Ipsilateral axillary nodal involvement has been shown to be present in more than 40 % of patients with primary BL, although this was seen in only 3/19 (16 %) of our cases. The size of primary BL lesions range from 1 to 5 cm in Yang et al.’s series with secondary BL tending to be smaller in size and more often multiple [6, 8, 10, 11].

### Ultrasound

The sonographic features of BL are variable. The most frequent appearance of BL at ultrasound is of a hypoechoic, irregular mass with indistinct margins (Fig. 1) [2, 8]. Interestingly, in the series reported by Surov et al. [6], approximately half of all cases demonstrated circumscribed margins. Heterogeneity of echo texture with hyperechoic regions, particularly a thick echogenic rim, may be also seen. This predominantly echogenic appearance was noted in approximately 23 % of our cases (Figs. 2 and 4). Lesions mimicking cysts were seen in four of our cases demonstrating marked hypoechogenicity and posterior acoustic enhancement. BL masses may have no internal vascularity seen on colour Doppler evaluation in up to 55 % of cases [6]. However, Doppler analysis was helpful to differentiate BL from a cyst in a case of an anechoic mass (Fig. 3). No posterior acoustic phenomenon was seen sonographically in 64 % of cases in the series of Yang et al. [8]; however, Liberman et al. [3] found posterior acoustic enhancement in 71 % (5/7) of cases. Posterior acoustic shadowing is not a feature of BL and this may be due to lack of desmoplastic reaction [12]. Elastography was performed in one case from our series. The mass was soft and was smaller on the elastogram than on B-mode imaging suggestive of a benign lesion. This is most likely due to the highly cellular nature with lack of desmoplastic reaction in some types of lymphoma. Three patients only had axillary adenopathy identified sonographically in our series.

### PET/CT

PET/CT has been shown to be useful in the staging of lymphoma with a sensitivity and specificity close to 100 % [13]. This technique is useful in assessment of treatment response demonstrating residual metabolically active tumour and areas of necrosis and fibrosis [13]. FDG PET can also be useful in female patients with suspected lymphoma with dense breast tissue which may obscure masses mammographically and on CT [5, 13]. Uptake on PET is usually homogeneous and intense with a reported average uptake value of 10.6 (Figs. 1 and 2) [7, 8] A “ring-shaped” peripheral pattern of hypermetabolism is sometimes encountered, particularly in rapidly growing or large tumours with areas of central necrosis or haemorrhage. If there is diffuse lymphomatous infiltration, then there may be associated hypermetabolic skin thickening which can resemble inflammatory breast carcinoma [7]. Hypermetabolic lymphadenopathy in the axillary, hilar regions or adenopathy elsewhere in the body may be a prominent finding in secondary lymphoma. Primary breast cancers including inflammatory breast cancer and metastases, especially from melanoma, may be difficult to differentiate from haematopoietic neoplasms. There may be a false-negative scan if the lesion is less than 1 cm [6].

## Breast leukaemia

Breast involvement with leukaemia is rare, with fewer than 200 cases reported in the literature [14, 15]. Both myeloid leukaemia and lymphocytic leukaemia involving the breast have been described, with acute myeloid leukaemia (AML) being most common [14]. Chronic myelogenous leukaemia (CML) and CLL in the breast are exceedingly rare [14]. Leukaemic involvement of the breast may be seen either in isolation or in the setting of widespread disease, and may be seen in the setting of leukaemic relapse [14–17]. The median age has been reported as 33 but with a wide age range of 1–80 years [14]. Patients may present with unilateral or bilateral breast masses with or without axillary adenopathy [14]. In our series there were 8 patients with leukemic involvement of the breast: 6 with chronic lymphocytic leukaemia and 2 with acute leukaemia. The unexpected predominance of chronic over acute leukaemia in our series may be due to the fact that in the clinical setting of acute leukaemia, breast masses are presumed to represent systemic manifestation of the disease and breast imaging and biopsy are not obtained if masses resolved with treatment.

### Mammography

**Fig. 5:**
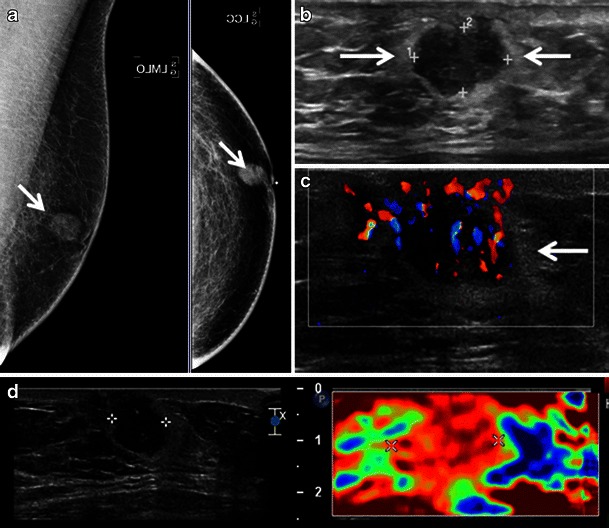
A 73-year-old man with a history of CLL and a new palpable mass in the left breast with associated nipple retraction. Biopsy showed chronic lymphocytic leukaemia. **a** Left breast medio-lateral oblique and cranio-caudal mammograms show an oval circumscribed subareolar mass with mild nipple retraction (*arrows*). **b**, **c** Ultrasound demonstrates an irregularly shaped, hypoechoic mass with angular margins and a thick echogenic rim which shows increased vascularity on colour Doppler evaluation (*arrows*). **d** Strain elastography (*red* equating to hard) demonstrates a hard mass which is significantly larger on the elastogram. The ratio of size was 1.99 between the B-mode and strain image.

Just over half of cases in one series had solitary lesions identified with the median size of lesion being 3.5 cm (range, 0.6–11 cm) [14]. Breast leukaemia commonly presents mammographically as a hyperdense mass with microlobulated margins. Microcalcifications are rare. Diffuse infiltration or architectural distortion may also be seen. In our series, half of the patients with CLL (3/6) had a mammographically detected mass; one case had a single circumscribed mass (Fig. 5) and the other two had multiple microlobulated masses. CLL was either mammographically occult or was not included in the mammographic field of view in the other three patients. Both of the patients with acute leukaemic involvement of the breast in our series presented with two or more masses in one or both breasts. Mammographically, one case showed multiple areas of architectural distortion (Fig. 7) and the other case showed multiple bilateral masses with irregular shape and indistinct margins.

### Ultrasound

**Fig. 6:**
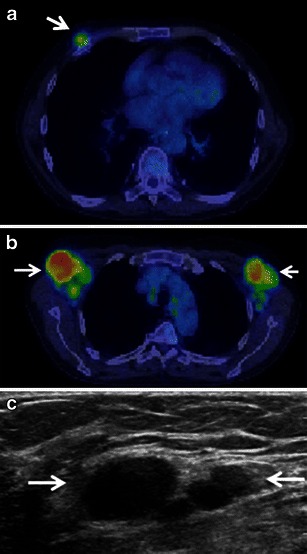
A 76-year-old man with known CLL presents with new palpable mass in the medial right breast which could not be included on a mammogram. Biopsy showed CLL. **a** Fused PET/CT image shows a hypermetabolic mass corresponding to the palpable mass in the medial right breast. **b** Fused PET/CT image demonstrates hypermetabolic axillary adenopathy (*arrows*). **c** Ultrasound shows a cluster of multiple hypoechoic masses with microlobulated margins without increased through transmission (*arrows*)

**Fig. 7:**
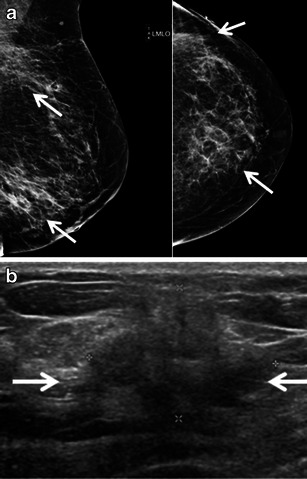
An 83-year-old woman with known acute T-cell prolymphocytic leukaemia presents with new palpable masses in the left breast. **a** Left medio-lateral oblique and cranio-caudal diagnostic mammogram demonstrates two areas of architectural distortion corresponding to the two palpable masses (*arrows*). **b** Ultrasound shows ill-defined mixed echogenic and hypoechoic mass (*arrows*). The other palpable mass had a similar sonographic appearance (*not shown*)

Sonographically, breast leukaemia may present as single or multiple heterogeneous hypoechoic masses. Of the cases where margins were reported, the majority had indistinct or microlobulated margins [14, 16]. Breast lesions were sonographically visible in all six of our patients with CLL, with all cases demonstrating hypoechoic masses; half of which were circumscribed (Fig. 6) and the other half demonstrating irregular shape and angular margins (Fig. 5). Increased vascularity was noted on colour Doppler interrogation (Fig. 5). Breast elastography was performed in one case. The tumour was hard on strain elastogram and was larger than on the B-mode image, suspicious for malignancy (Fig. 5). Axillary adenopathy was noted in two cases. In both patients with acute leukaemia, masses demonstrated irregular shape and non-circumscribed margins and heterogeneous echogenicity with a significant echogenic component (Fig. 7). No axillary adenopathy was seen in either patient with acute leukaemia.

### PET/CT

Ginat et al. [7] noted that leukaemic deposits in the breast present as hypermetabolic breast masses on PET/CT. Hypermetabolic axillary adenopathy can also be appreciated as seen in our two patients (Fig. 6).

## Multiple myeloma

Multiple myeloma represents one of the plasma cell malignancies, neoplasms with unabated proliferation of malignant plasma cells [17]. Extramedullary plasmacytomas occur most often in the head and neck region, but may also occur in many other organs including skin, lungs, gastrointestinal tract and bladder [9]. Similar to the other haematological diseases with mammary involvement, multiple myeloma in the breast is very rare [9, 17, 18]. In the breast, myeloma most often presents as an extramedullary manifestation in a patient with known bone marrow involvement. Less often, breast involvement with myeloma occurs in the absence of marrow disease, referred to as breast plasmacytoma (BP). Primary BP is exceedingly rare, accounting for less than 1 % of all extramedullary plasmacytomas [19]. The mean age of presentation is 55 years. Approximately 20 % of patients who develop an isolated breast plasmacytoma later develop multiple myeloma [9].

### Mammography

**Fig. 8:**
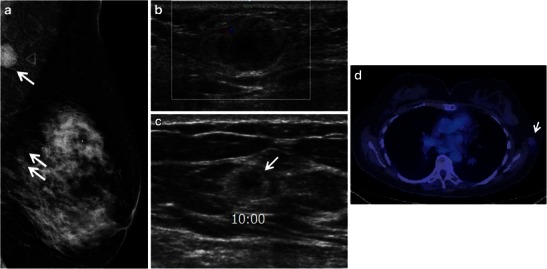
A 57-year-old woman with known multiple myeloma of the sacrum and multifocal involvement of the left breast and low axilla. **a** Full-field digital medio-lateral oblique view of the left breast demonstrates a round, circumscribed 1.9-cm mass (*arrow*) in the upper outer left breast/low axilla corresponding to a palpable abnormality. Two additional circumscribed masses were noted in the superior left breast posterior depth (*arrows*). **b** Ultrasound of the palpable low axillary mass imaging demonstrates a 2.0-cm heterogeneous mass with circumscribed margins with only mild increase in vascularity on colour Doppler. **c** Ultrasound of the mass at the 10 o’clock position of the left breast shows a microlobulated principally echogenic mass with central area of hypoechogenicity (*arrow*). The mass at the 9 o’clock position of the left breast had a similar sonographic appearance (*not shown*). Ultrasound guide core biopsy of all three lesions showed multiple myeloma. **d** Minimal FDG uptake is noted within the left upper outer/low axillary mass (*arrow*) on PET/CT

The imaging appearance of myeloma in the breast is not well documented in the literature, conveyed primarily through case reports [20–24] and a recent review of the literature by Surov et al. [20]. Primary BP demonstrates a similar imaging appearance to cases of secondary extramedullary breast involvement in patients with known bone marrow disease [20]. A round, oval or lobulated mass is noted at mammography in the majority of patients (Fig. 8). Margins are well defined in approximately one-half of all cases. Less common mammographic presentations include diffuse involvement of the breast with an infiltrative mass or less often a negative mammogram. Multiple masses are seen in approximately one-third of cases (Fig. 8).

### Ultrasound

At ultrasound, myeloma in the breast is most often homogeneously hypoechoic, much less likely hyperechoic or of mixed echogenicity (Fig. 8) [20, 25]. Margins are well defined by ultrasound in approximately one-half of all cases.

### PET/CT

As with the other haematological malignancies, PET is useful for staging purposes and assessment of treatment response. On PET/CT, breast myeloma masses are often well defined and demonstrate relatively low-grade FDG uptake (Fig. 8) [7].

## Conclusions

Haematological malignancies of the breast are rare. They demonstrate a variable imaging appearance but most frequently manifest as lobular or irregular mass(es) with indistinct margins at mammography and solid, irregular hypoechoic masses at ultrasound. MRI typically shows plateau kinetics and there is usually avid homogeneous hypermetabolism on PET/CT with the noted exception of myeloma. In the appropriate clinical context, these imaging findings should help alert the radiologist to the correct diagnosis and ultimately prevent unnecessary surgical treatment.
